# Land-use induced soil carbon stabilization at the expense of rock derived nutrients: insights from pristine Andean soils

**DOI:** 10.1038/s41598-023-30801-x

**Published:** 2023-03-20

**Authors:** Hermann F. Jungkunst, Felix Heitkamp, Sebastian Doetterl, Steven P. Sylvester, Mitsy D. P. V. Sylvester, Vanessa Vetter, Shafique Maqsood, Thorsten Zeppenfeld, Michael Kessler, Sabine Fiedler

**Affiliations:** 1 iES Landau, RPTU Kaiserslautern-Landau, Fortstraße 7, 76829 Landau, Germany; 2 Environmental Control, Northwest German Forest Research Institute, Grätzelstrasse 2, 37079 Göttingen, Germany; 3 Department of Environmental Systems Science, ETH Zurich, Zurich, Switzerland; 4 Department of Systematic and Evolutionary Botany, University of Zurich, Zollikerstrasse 107, CH-8008 Zurich, Switzerland; 5 Department of Soil Science, Faculty of Organic Agricultural Sciences, University of Kassel, Steinstrasse 19, 37213 Witzenhausen, Germany; 6 Forest Growth, Northwest German Forest Research Institute, Grätzelstrasse 2, 37079 Göttingen, Germany; 7 Institute of Geography, Johannes Gutenberg-University Mainz, Johann-Joachim-Becherweg 21, 55128 Mainz, Germany

**Keywords:** Carbon cycle, Biochemistry

## Abstract

Soils contain significantly more carbon than the atmosphere, hence we should understand how best to stabilize it. Unfortunately, the role of human interventions on soil organic carbon (SOC) persistence in the Anthropocene remains vague, lacking adequate sites that allow unbiased direct comparisons of pristine and human influenced soils. Here we present data from a unique study system in the High Andes that guarantees pristineness of the reference sites by physical inaccessibility through vertical cliffs. By comparing the isotopic signatures of SOC, mineral related carbon stabilization, and soil nutrient status across grazed versus pristine soils, we provide counterintuitive evidence that thousands of years of pastoralism increased soil C persistence. Mineral associated organic carbon (MAOC) was significantly higher in pastures. Land use increased poorly crystalline minerals (PCM’s), of which aluminum correlated best with MAOC. On the other hand, human’s acceleration of weathering led to acidification and higher losses of cations. This highlights a dilemma of lower soil quality but higher persistence of SOC due to millennia of pastoralism. The dynamics of soil genesis in the Anthropocene needs better understanding, but if human-induced weathering proves generally to promote soil carbon persistence it will need to be included in climate—soil feedback projections.

## Introduction

Humans dominate soil development in the ongoing Anthropocene^1–4^ but resulting biogeochemical processes are yet to be fully understood. There are examples of positive human influence on soil organic carbon (SOC) storages most prominently Terra Preta, and Plaggic or Hortic Anthrosols^5,6^. However, process understanding of carbon (C) persistence in soils remains one of the greater challenges to understand anthropogenic modifications adding to climate change science^7,8^. To separate human influences from other factors, a comparison to pristine counterparts is required, but these barely exist on our planet^2^. The alternative of studying processes happening in soils that have been taken out of use leads to different insights. What is needed, then, is the ability to directly compare pristine, reference sites with sites that have been influenced by historical human activities like grazing. Such a unique study system exists in the High Andes, where intensive studies have shown that soil-forming processes would have played out similarly in the accessible grazed versus inaccessible pristine sites in the absence of pastoralism^9,10^. Soils formed from very similar parent material and erosion was ruled out. Physical inaccessibility by vertical cliffs guaranteed that pristine sites were never grazed, therefore any differences in soil properties, like lower exchangeable cation nutrients, could be related to human influence by grazing^9,10^. Like in most other studies total C stocks of pasture soils did not differ significantly from soil C of comparably less disturbed forest stands but were even higher^11^. However, carbon quality or persistence, the cause for relatively high carbon stocks under pastures, was yet to be studied in that unique Andean system. To unravel the reasons for pasture soils holding about as much C as forested sites is of global interest, as pastoralism has been credited with sustaining humanity since Neolithic times and is still practiced across approximately 25% of the Earth’s land area^12^ Furthermore, mountain ecosystems hold an estimated share of 29% of C stored globally in soils^13^. For the first time to our knowledge, the groundwork has been laid to study the millennial-scale human influence of globally relevant pastoralism on soil C persistence.

Our previous work^9^ revealed that thousands of years of pastoralism accelerated soil weathering. The investigated soils were shallow (no B-horizon developed), acidic, and poor in clay but strictly autochthonous. Contrary to intuition, grazed sites showed higher bulk C values than their pristine counterparts. This finding is important, as SOC plays a fundamental role in soil quality by storing nutrients and improving aeration and water-holding capacity^14^. However, exchangeable cation nutrients were much lower in managed soils and therefore human extractions must outweigh the general storage capacities of soil C. The managed soils also differed from pristine soils in PCM’s (poorly crystalline minerals) and exchangeable Al, but not in pH. Apparently, advanced weathering under pasture was the driving force behind these findings. Since Slessarev et al.^15^ nicely showed how weathering controls the potential for soil carbon storage at a continental scale, pasture induced weathering could be causal for C stabilization in soils. Rasmussen et al.^16^ and several subsequent studies^17–19^ revealed that PCM’s, extracted by oxalate, are a cause for the control over organic C storage in non-arid, acidic soils. Mineral associated organic carbon (MAOC), which includes metal-C associations, protects SOC from decomposition by limiting microbial access in biologically non‐preferred soil spaces^20,21^. Building upon theses finding and our previous studies, we were consistently taking the next step, by now analyzing indicators for soil carbon stabilization. Hence, for this study we measured multiple indicators of SOC stability as well as specific N and P values for traditional pastures of the high Andes (managed pastures, MP) and pristine High Andean ecosystems composed of mosaics of forest (pristine forests, PF) and grasslands (pristine grasslands, PG) and related them to weathering products. We hypothesized that soil C persistence is higher in the managed soils due to the stabilization of C in mineral associated form by PCM’s. As we expected soil C to be bound more to PCM’s, we predicted other soil quality indicators that are associated to organic matter such as soil nitrogen (N) and phosphorous (P) also to be higher in managed soils.

## Results

### Soil carbon persistence

**Figure 1:**
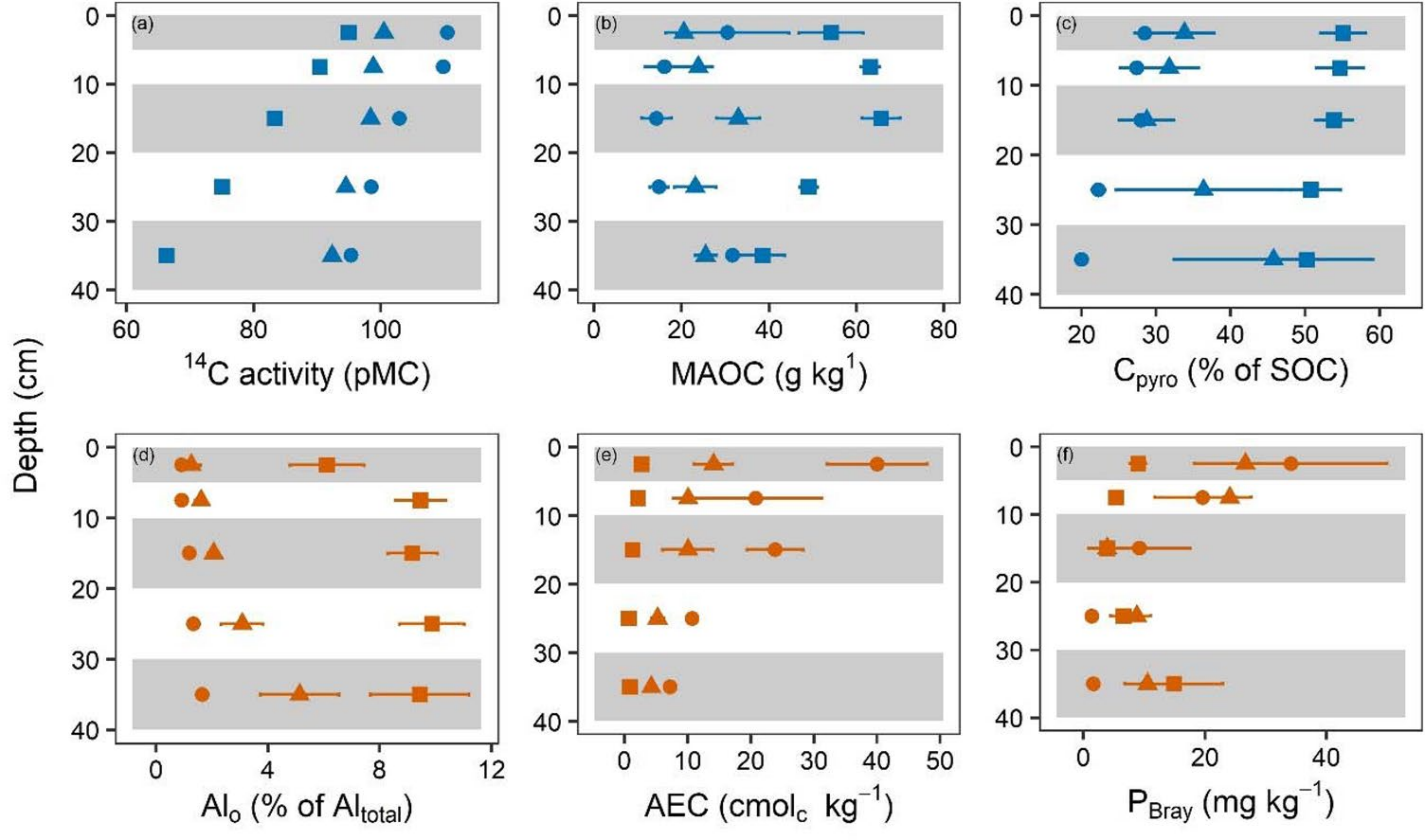
Depth profiles of selected soil carbon persistence indicators (blue) and soil quality indicators (orange). Managed pastures (MP) are displaced as squares and pristine sites as triangles (grassland, PG) and circles (forest, PF). Mean values and standard errors (b-f; n = 3). (**a**) The fraction of modern carbon indicates the apparent ^14^C age of the soil carbon associated with fine (< 63 µm) mineral phase (only for this parameter one profile (otherwise three profiles) per location was measured and measurement errors are too small to be seen); (**b**) the soil carbon associated with the fine mineral phase (C_<6 µm_, MAOC); (**c**) pyrophosphate extractable carbon (C_pyro_) as a measure for soil organic carbon persistence. (**d**) oxalate extractable Aluminum (Al_o_) as indicator for soil weathering; (**e**) alkaline and exchangeable cations (AEC) representing soil fertility and (**f**) exchangeable P (P_Bray_) an anion to complement soil fertility indication.

The sum of our analyses of SOC quality and quantity (Supplement Information SI-1) strongly point towards an enhanced persistence of SOC under pastoralism. Fine root mass (kg m^-2^, means ± standard error) in managed pastures (1.5 ± 0.3) was significantly lower than in PG (2.8 ± 0.6) and PF (4.8 ± 0.5). Total SOC stocks (kg m^−2^) revealed an opposing trend, as pastures had the highest values (21.6 ± 0.6) and the two pristine sites showed similar values (PG: 15.3 ± 3.5, PF: 16.1 ± 3.2). Although the differences were not significant, this contrasts common expectation that removal of C input by management leads to reduction of soil C stocks^22^. In our analysis, soil C persistence in pristine and managed sites is expressed as marked differences in soil C age. For MAOC (C in the soil fraction < 63 µm), we found lower ^14^C throughout all depths of the MP profile (Fig. 1a), indicating older SOC in pastures than in pristine sites. Differences in ^14^C between MP and pristine sites increased with depth, and PF and PG were more similar than PG and MP. Consequently, the influence of land-use clearly overruled the influence of vegetation type. This applied to all measured variables. Soil C persistence ideally manifests in older but less decomposed organic C. We used isotopic δ^13^ signature of C as an indicator of the degree of decomposition^23,24^. The δ^13^C (‰) of the C sources (root material) varied (PG: − 26.57 ± 0.14, PF: − 27.44 ± 0.21, MP: − 25.83 ± 0.10), so the difference (delta) between plant root material and MAOC was used. The degree of decomposition was lowest in MP, as the Δδ^13^C signal (‰) of PF and PG had an enrichment of 1.7–2.8, whereas MP was enriched by only 0.9–1.5. Hence, SOC of the managed pasture was older but less decomposed. Decomposition is linked to the energy budget of microorganisms^25^. The resistance against thermal oxidation simulates the activation energy needed to decompose SOC. Temperatures needed to oxidize 50% of total C (T50, °C)^26^ were significantly higher in pasture soils (344 ± 2.2) than in pristine sites (PG: 288 ± 13, PF: 297 ± 2). Therefore, we conclude that energy investment by microorganisms to decompose organic compounds is higher in pastures than in pristine systems, which explains the paradox of older age and lower decomposition rate. This interpretation is supported by the significantly low storage of microbial C (% of SOC) in MP (0.62 ± 0.09) as compared to PG (1.97 ± 0.26) and PF (1.75 ± 0.19). Moreover, specific microbial respiration (qCO_2_, mg CO_2_-C kg^−1^ C_mic_^−1^ day^−1^) was three to four times higher in MP (115.5 ± 5.6) as compared to PG and PF (21.3 ± 7.5 and 27.2 ± 3.1, respectively) (SI-2), indicating high energetic investments to maintain or build up biomass.

**Figure 2:**
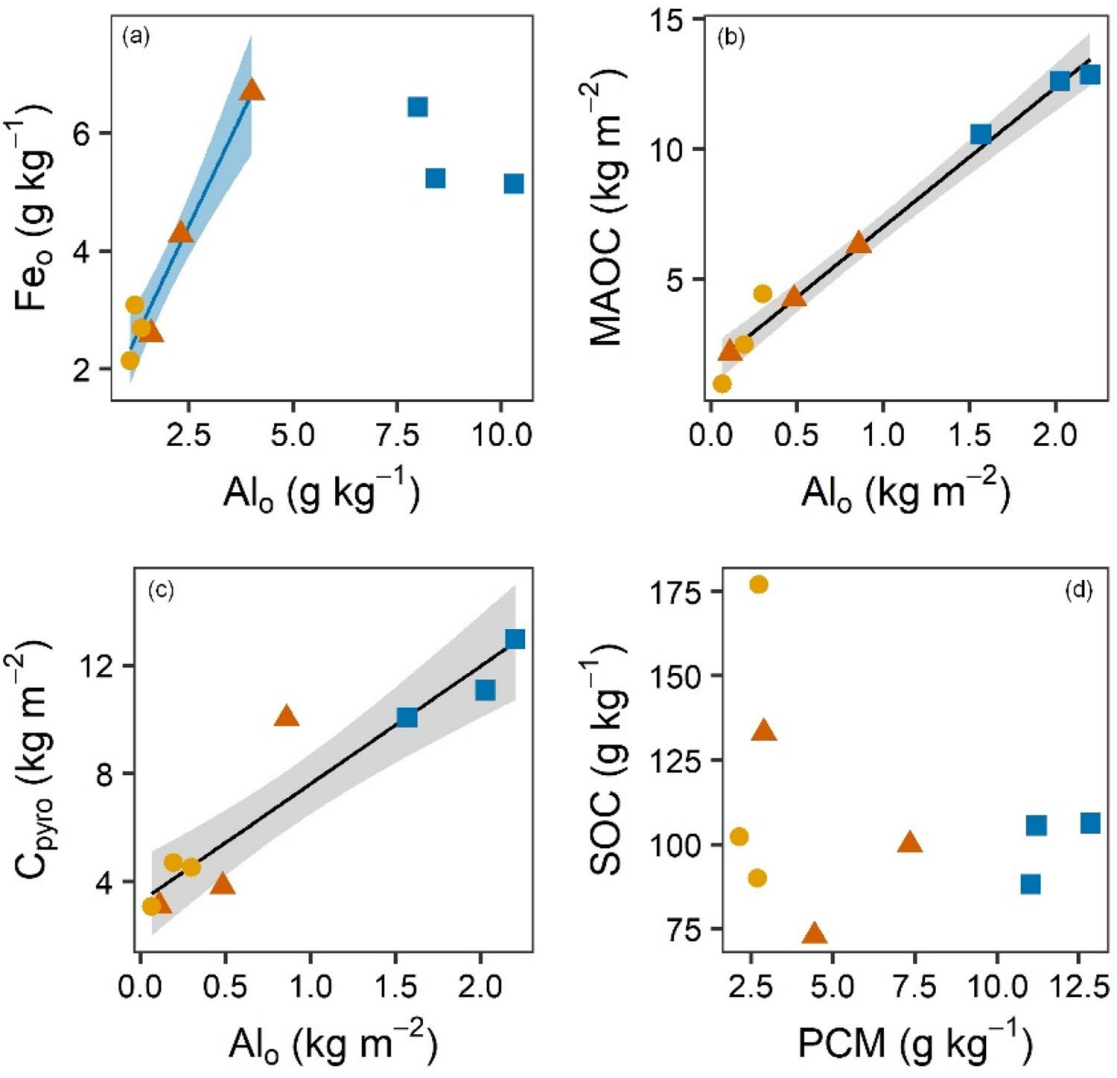
Relationships between (**a**) oxalate extractable iron (Fe_o_) and aluminum (Al_o_), (**b**) mineral associated organic carbon (MAOC) and oxalate extractable aluminum (Al_o_), and (**c**) pyrophosphate extractable carbon (C_pyro_) and poorly crystalline minerals, and (**d**) total soil organic carbon (SOC) and poorly crystalline minerals (PCM). Concentrations given as the soil mass-weighted mean of depth increments of individual profiles, stocks are the sum of stocks of the depth increments. Squares: managed pasture (MP), triangles: pristine grassland (PG), circles: pristine forest (PF). Shaded area indicates the 95% confidence interval of the linear model fit.

To localize soil C persistence, a density fractionation was applied to isolate C associated with the mineral fraction. The mineral fraction was additionally separated into > 63 µm (sand) and < 63 µm (silt and clay). The amount of MAOC (C associated with the fine mineral fraction i.e. < 63 µm) was markedly higher throughout the depth increments of the MP profiles compared to PG and PF soils (Fig. 1b). In sum, meaning per soil pit, MP had more than double the amount of MAOC in % of SOC as compared to PG and PF (55.7 ± 2. vs. 26.9 ± 1.8 and 15.2 ± 3.6). However, due to weathering^9^, silt and clay content (kg m^-2^) were higher under pastoralism (87.5 ± 9.9) than under natural conditions (PG 50.3 ± 13.4, PF 36.8 ± 14.8). These differences potentially explain the variation in C associated with the fine mineral fraction. However, the differences found per total mass unit soil were maintained per mass unit fine mineral fraction (g C kg^-1^ fraction^-1^) (130.3 ± 7.1 in MP vs. 88.1 ± 8.7 and 76.0 ± 1.0 in PG and PF). Consequently, higher silt and clay contents only partly explained higher C contents in managed soils. Therefore, the amount of MAOC was significantly higher in pastures, but it was not necessarily less accessible to microbial decomposers. Pyrophosphate extractable C was used to assess the degree of inaccessibility and therefore acted as a measure for SOC persistence. In that sense, SOC persistence was about twice as high in pasture profiles varying with depth (Fig. 1c). In total, this translates to 52.6 ± 2.4% of SOC in the managed soil belonging to the more persistent pyrophosphate extractable C fraction, compared to only 26.6 ± 2.7 to 35.3 ± 6.3% of SOC in natural soils. Together, these results explain the higher C storage of the managed soils and clearly show that millennia of pastoralism led to a higher persistence of SOC. Weathering products most likely drive higher SOC persistence in managed soils^15^. But, as to be expected at the elevation and climate of our study area^9^, weathering and particularly soil development were slow. Nevertheless, silt content, and Al_o_ were significantly higher in managed sites (Fig. 1d), but Fe_o_ only insignificantly higher. For the pristine sites we observe a striking linear relationship between Al_o_ and Fe_o_ (r^2^ = 0.98) which completely vanished for the managed sites (Fig. 2a). This is a clear hint, that land use does not simply change the intensity or speed of weathering but alternates processes. Nevertheless, an almost perfect linear relationship (r^2^ = 0.98) is identified when the mass of C in the fine mineral fraction of the whole profile is plotted against the mass of Al_o_ (Fig. 2b). Since this relationship is similar but not improved by using the standard PCM (1/2 Fe_o_ plus Al_o_; r^2^ = 0.98) (not shown), it is concluded that Al_o_ is controlling soil carbon persistence (as measured in C_pyro_ vs. PCM in Fig. 2c). Relationships probably will differ across ecozones^27^ but potentially explaining variables always will proof independent of land-use. Most likely for low soil development (no B-horizon) exhibiting a high amount of unassociated carbon (POM) the standard relation between PCM (g kg^−1^) and total SOC (g kg^−1^) will not result in tight relationships like for this study (Fig. 2d).

### Soil quality

Despite similar pH values (4.2–4.7), the Al-saturation, i.e., portion of exchangeable Al on total effective cation exchange capacity (CEC), was significantly higher in MP, pointing towards low remaining proton buffering capacity in MP. Hence, the crossing of a tipping point for proton buffering is expected in MP much earlier than in the pristine sites. Most likely tipping points have been crossed or occurred in linear losses for alkaline exchangeable cations (Ca, Na, K, Mg) in the managed soil (Fig. 1e). The CEC (mol_c_ m^2^) was significantly lower in MP (140 ± 5) than in PG (180 ± 23) and PF (296 ± 69). Humans generally increase leakages in nutrient or biogeochemical cycles by extracting livestock biomass from pastures. Moreover, burning, which further promotes nutrient losses, is a common management tool in these landscapes. However, no significant differences were found for N and P. Significantly higher legume abundances on the managed sites, which were nearly absent on pristine sites (Sylvester et al. unpublished data), may have refilled N losses by increased atmospheric N-fixation. The isotopic signature of N was measured as an indicator for the openness of the N cycle^28^. We found that the δ^15^N (‰) of MP was markedly higher (6.13 ± 0.34) compared to pristine sites (3.78 ± 0.96 and 2.71 ± 0.42 in PG and PF, respectively). This shows that losses of ^14^N from MP are higher compared to pristine sites. Nitrogen in microbial biomass is a measure of the availability of organic N. Microbial biomass N (% of total N) was more than four times higher in pristine sites (PF 4.3 ± 0.4, PG 4.1 ± 0.7) than in MP (1.0 ± 0.2) and correlated with δ ^15^N (r_pearson_ = − 0.75, p = 0.02). Hence, pasture soils show increased N losses and leakages in N cycling and a lower availability of N compared to their pristine counterparts, which, however, did not influence N stocks. Bioavailable phosphorous (P_Bray_) was not significantly different when summed up per soil profile. However, significant differences were found in the depth distribution of P_Bray_ (Fig. 1f). In the upper 10 cm, P_Bray_ was significantly lower in MP and was partly compensated by higher values in the deepest layer (Fig. 1f). Latter fits to the expectation that rock weathering leads to increased P availability^29^. In summary, the macronutrients N and P neither mirrored nor counterbalanced the significant lower values of the alkaline nutrients (Ca, Na, K, Mg) (Fig. 1i) in the managed soil. Therefore, grazing reduced soil quality.

### Structural equation model (SEM)

**Figure 3:**
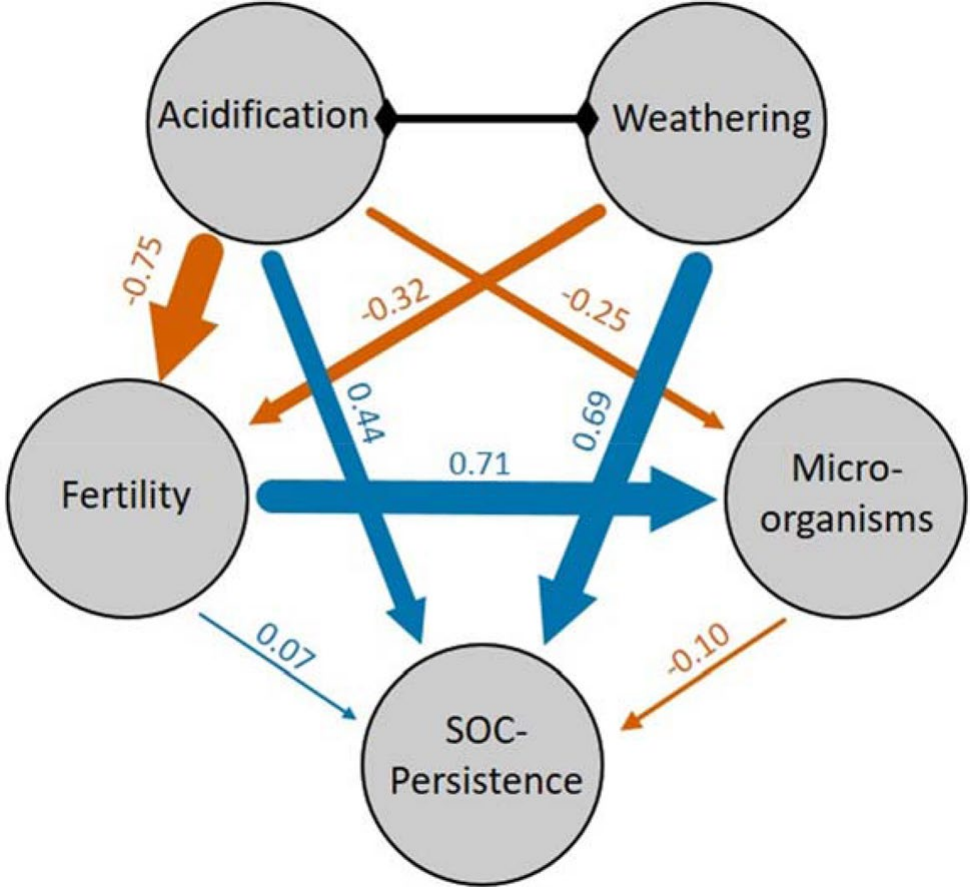
Median effect sizes between the latent variables of the structural equation model. Blue arrows indicate positive, orange arrows negative relationships. Acidification and weathering were assumed to correlate. Arrow thickness is proportional to effect size, which is given by the numbers. The latent variables were loaded with manifest (i.e. measured) variables (Table S1).

We elucidated direct and indirect functional interdependencies with multivariate structural equation modelling^30^. Latent constructs (acidification, weathering, fertility, microorganisms, and SOC persistence) were defined by a set of measured variables. Acidification and weathering were assumed to be interdependent. The clearest result was a negative effect (Effect Size, ES − 0.75, 95% credible interval: − 1.74 to 0.30) of acidification on fertility, whereas fertility was positively related (ES 0.71, 95% credible interval: 0.26 to 1.22) to microorganisms (Fig. 3). Both weathering (ES 0.69, 95% credible interval: − 0.17 to 1.59) and acidification (ES 0.44, 95% credible interval: − 0.61 to 1.50) affected SOC persistence positively. This finding corroborates the results discussed above, although uncertainty of the effects was high. While the combination of advanced weathering and higher acidification under MP led to higher stabilization of soil carbon, acidification also drove a marked decline in soil fertility.

## Discussion

Multiple individual indicators from our study and the SEM framework show that thousands of years of pastoralism led to higher, older, and less decomposed SOC stocks, but also to less fertile soils. Further, our findings support the organo-metallic glue hypothesis by Wagai et al.^31^. Organo-metallic liaisons increase soil C persistence by limiting microbial access^32^. In our study system, weathering supports soil C persistence at the expense of soil quality^14^. It is striking that the differences between managed and pristine soils clearly outweigh differences between natural forest and natural grassland soil. With this work we present for the first time statistically corroborated data on the effect of long-term pasture management on carbon stabilization in relation to weathering. Causes for human accelerated weathering due to grazing must still be unraveled but may relate to increased proton input by animal excrements and plants to receive rare cations.

Our understanding of the importance of the mineral matrix to stabilize soil carbon has been growing in recent years^16–19,33^. Slessarev et al.^15^ generally revealed that weathering controls the potential for soil carbon storage. Doetterl et al.^34^ using a chronosequence approach, argued that mineral assemblage, driven by weathering, is the main factor explaining variation in SOC-stocks over time. The human impact on weathering was mainly attributed to land conversion-induced soil erosion, which brings the weathering front closer to the surface. Here, we expand this view on the human impact on weathering by showing that land conversion also directly affects weathering by changing microclimate^9^ and inducing acidification. The link between mineral weathering and land use-induced acidification has to be taken into account due to the impact on soil fertility. Decreases in soil fertility are linked to primary productivity, thus C-input to soil. Such decrease in C-input is not necessarily accompanied with decreasing C-stocks. In our case study, it is likely that C-stocks decreased markedly shortly after conversion of the woodland mosaic to pasture (“fast out”)^35^, because C-stocks in natural, less weathered soils are mainly input driven. Along the trajectory of soil development with increased weathering speed in pasture soils, C-stocks became stabilization-driven (“slow in”)^35^. From an anthropocentric point of view, this is good, as stabilized C is not as vulnerable to changing climate as is labile C fractions in soil. However, this increased stability against climatic effects (including variation in C-input) comes at the cost of soil fertility with consequences for livelihood of local populations. Moreover, the future trajectory of soil development must be studied. It was shown that highly weathered soils loose much of their capability to store carbon as further enhanced weathering leads to the reductions of minerals with highly reactive surfaces^36^. The increased weathering speed in pasture soils implies that this tipping point, where C-stabilization will not counter act low C-inputs may come earlier. Therefore, human-induced weathering most likely promoted the formation of MAOC and has the potential to increase soil carbon persistence for low developed soils but not for highly developed soils. We conclude that the influence of humans on the relationships between organic and mineral matter in the soil, which exists now for thousands of years, is extremely important and needs to be better understood to improve climate—soil feedback understanding.

## Materials and methods

### Environmental setting

The study site, locally called ‘Cancha Cancha’, is situated in the Cordillera Urubamba in Cusco province, southern Peru, 30 km north of the town of Urubamba (13°14′35’’S; 72°10′18’’W) at 4500 m above sea level (a.s.l.). The site is in the puna belt within the ‘tierra helada’ which is characterized by low annual mean temperatures, a high diurnal temperature amplitude and a semi-humid climate with a pronounced dry season from May to October^37^. Mean annual precipitation in the closest climate stations ranges from 454 mm (Urubamba, 2863 m a.s.l.) to 1606 mm (Winaywayna, 2800 m a.s.l.)^38^. Since our study site lies 1600 m above the climate stations, the data are hardly comparable.

Nevertheless, orography and vegetation pattern in Cancha Cancha indicate that mean annual precipitation ranges at the higher end of these records. The vegetation is classed as ‘Puna’ and is characterized by extensive tracts of tussock grassland interspersed with patches of *Polylepis spp.* (Ruiz & Pav.) forest.

### Selection of study sites

The study took place on two main situations. The accessible site is accessible for the local population and received considerable pressure by grazing (camelids and later on sheep) for presumably for several millennia^9,39^. The pristine site is surrounded by steep cliff, only accessible with mountaineering equipment. This ledge has a size of ca. 0.2 km^2^ and therefore large enough to represent a comparable situation to the accessible site regarding landscape position and soil development. Both sites were never directly glaciated, as they were above the shoulders of a U-shaped valley. The vegetation of the accessible site was typical for the grazed puna biome and consisted mainly of herbaceous taxa. Single trees (*Polylepis*. spec.) existed, but no closed forest was found. The pristine vegetation consisted of a mosaic of grassland and *Polylepis* forest with trees up to 5 m height.

Nine soil profiles were dug down to the bedrock, with a maximum distance of 500 m between each other. Three profiles represented managed pasture (MP) on the accessible site. Six profiles were dug on the inaccessible site, three of each representing pristine forest (PF) and pristine grassland (PG). All profiles shared comparable exposition (290° to 310°). A different slope angle was chosen for each replicate with the classes “strongly sloping” (10–15%), “moderately steep” (15–30%), and “steep” (30–40%). Statistical analysis with a linear mixed model with “slope” as random factor revealed no effects of the slope angle and therefore slope was dropped from the subsequent analysis. Despite the steep terrain, there were no visible sites of erosion. This was possibly prevented by the dense vegetation on pristine sites (cover > 96%) and by a dense network of small animal paths which served as slope-parallel terracing. The soil surface on the paths was intact as sheep and camelids do not wound the soil as e.g. cows.

Bedrock was classified as the vulcanite andesite. There was concern that soils did not develop in situ, as mass movements or deposition of volcanic ashes were possible. However, using rare earth elements of bedrock and soil as geochemical markers^9^ proved in situ genesis of soil. This finding was further corroborated by a similar depth gradient of coarse fragments in all profiles.

### Sampling and sample preparation

Sampling and sample preparation procedures were described in detail in Heitkamp et al.^9^. Briefly, three pits for each vegetation type, pristine forest (PF), pristine grassland (PG), and managed pasture (MP) were dug. Volumetric samples were taken by using three cores (100 cm^-3^ each) or, in case of high rock content, by the volume replacement method. Depth increments of 0–5, 5–10, 10–20, 20–30 and 30–40 cm were used. Samples were sieved at field moisture (≤ 2 mm), and roots and coarse fragments were washed, dried, and weighed. Bulk density of the volumetric soil samples was calculated from total field moist weight after correcting for water, coarse fragments, and root content.

Soil analysis followed the methods by van Reeuwijk^40^, unless stated otherwise. Considering bulk density, soil horizon thickness, stone content, and element concentration, we calculate the element mass of fine earth per square metre. When giving mean values of concentrations for the whole profile, depth wise values where weighted by the corresponding soil mass before calculation.

### Standard analysis

We determined particle size distribution by sieving and sedimentation. **pH** was measured in 0.1 m CaCl_2_ in a 1:5 soil:solution ratio (*v*/*v*). **Exchangeable cations**^41^ were determined by extraction with 1 m NH_4_Cl and Ca^2+^, K^+^, Mg^2+^, Na^+^, Al^3+^, Fe^3+^, H^+^, and Mn^2+^ were quantified by inductively coupled plasma—optical emission spectrometer (ICP-OES) (Optima 4300 DV, Perkin Elmer Instruments, Norwalk, USA). Effective cation exchange capacity (**CEC**) was calculated as the sum of the quantified cations. Total element concentrations were also measured by ICP-OES, after digestion with HNO_3_/HF^42^.

Fractions of Fe and Al were extracted with ammonium oxalate (**Fe**_**o**_** and Al**_**o**_). Element concentrations were measured by ICP-OES. “Fe- and Al-associated” soil organic carbon (SOC) were extracted with sodium pyrophosphate (**C**_**pyro**_), which targets organic matter bound to mineral surfaces via ligand exchange and cation bridging as well as organic matter in metal–organic matter complexes^43^. Carbon concentrations of the extracts were measured (Dimatoc, Dimatc, Essen, Germany).

The plant-available phosphorus (**P**_**Bray**_)^44^ in soil was extracted by a solution consisting of HCl, and NH_4_F, and analysed colorimetrically as molybdenum-blue-complex by spectroscopy (Lambda 40, Perkin Elmer, Waltham, USA) at 880 nm.

### Density-size fractionation

We carried out a combined density-size fractionation according to Dichon et al.^45^. Triplicate samples of 7 g were shaken for 20 min in 30 mL sodium polytungstate (SPT) with a density of 1.6 g cm^−3^ (20 °C). Afterwards, samples were put in an ultrasonic water bath receiving a total of 450 J mL^−1^ over the course of one hour. Samples were then placed in a centrifuge, allowed to adapt to the set temperature (20 °C) and centrifuged for 30 min (4000 g). The light fraction was decanted, and the process was repeated. The light fraction (ρ > 1.6 g cm^−3^) was filtered (< 0.45 mm) and rinsed thoroughly with distilled water. The heavy fraction (ρ < 1.6 g cm^−3^) was sieved (63 µm) to separate the sand and silt plus clay fractions. The latter was rinsed with water and vacuum filtered (< 0.45 µm) to remove remaining SPT. Clay (< 2 µm) and silt (63–2 µm) fractions were separated by sedimentation according to Stoke’s law. The obtained fractions were also used to determine particle size distribution, after correcting for organic matter content. We used a van Bemmelen conversion factor of 1.724^46^ to calculate organic matter from C-contents. The light and sand fractions were air-dried (40 °C), and the silt and clay fractions were freeze-dried after another filtration. All fractions were subjected to C and N analysis, as described below. Material recovery was 99.3% (standard deviation: 1.2%) and C– and N–recovery was 95% (standard deviation: 14%) There was no systematical difference in recovery between sites.

### Carbon and nitrogen measurements

**SOC and total N** of bulk soil and fractions were measured by dry combustion (950 °C, Truspec CHN LECO, St Joseph, MI, USA). Carbonates were not present in the acidic soils. Stable isotopes (^**13**^**C and **^**15**^**N**) were measured at the Center for Stable Isotope Research and Analysis, Göttingen, Germany by isotope-ratio mass spectrometry (IRMS) (Finigan MAT, DELTA^plus^) and expressed as δ^13^C values (‰ V-PDB) or δ^15^N values (‰ air). The difference of plant material and soil is given as Δδ^13^C values, thus more negative values indicate higher ^13^C enrichment in soil relative to plant material. Because light-fraction C can be “modern”, even in buried paleosols^47^, ^**14**^**C** was measured in fraction < 63 µm at the Max-Planck-Institute Jena, Germany by Accelerator Mass Spectrometry (AMS) (MICADAS, Ionplus, Dietikon, Switzerland) and results are expressed as percent modern C (pMC). ^14^C measurements were only performed on one profile per treatment. Thermal stability of soil C was determined as **T-50**, which is the temperature at which 50% of SOC evolved as CO_2_^48^. CO_2_ evolution was measured by infrared detection during ramped combustion (140–900 °C in 20 min) in a pure O_2_ atmosphere by a multiphase carbon determinator (RC-412, Leco, St. Joseph, MI, USA)^49^.

### Microbial biomass and respiration

Basal respiration was measured in the lab on pre-incubated (3 days) soil samples at 23 °C and 55% water holding capacity. Incubation lasted for a week with three periods of CO_2_ measurements (EGM-4, PP-Systems, Amesbury, USA). The metabolic quotient (**qCO**_**2**_) was calculated by standardizing respiration to microbial biomass C^50^. Microbial biomass was measured by the chloroform-fumigation extraction method after incubating the samples for 10 days^51^. Carbon (**C**_**mic**_) and nitrogen (**N**_**mic**_) were extracted with 0.5 m K_2_SO_4_ and measured in the extracts (Dimatoc, Dimatec, Essen, Germany). Conversion factors for extractable elements after fumigation to microbial biomass of 0.45 for C and 0.4 for N were used^52^.

### Statistics

Statistics were done for values of the whole soil profile (sum for stocks or mass-weighted means for concentrations), without consideration of depth increments. Significance of differences between mean values was tested by a one-way ANOVA followed by a Tukey HSD post-hoc test. Correlations were calculated with Spearman’s rank correlation coefficient. Analyses were done in R 3.6.2 using the packages “lme4” and “Hmisc”.

To elucidate functional interdependencies of pedogenic processes we applied a structural equation model (SEM). A set of structural equations was used to simultaneously model direct and indirect relationships among variables^30^. Variables constitute here quantified soil features (manifest variables) like H^+^ concentration. Further variables could be unobserved (latent) constructs which represent hypothesized underlying features like acidification, which in turn is explained by manifest variables like H^+^ concentration or Al^3+^ saturation (Table 1). In the causal network of SEM, the latent variables could act simultaneously as cause and response (Fig. 3). For example, “fertility” could be influenced by “weathering” causing at the same time an effect on “microorganisms”.

**Table 1:** Description of the composition of the latent variables.

Latent variable	Manifest variables	Calculation or unit
Weathering	Oxalate-extractable Al in relation to total Al	Al_o_/Al_total_
	Oxalate-extractable Fe in relation to total Fe	Fe_o_/Fe_total_
	Dithionite-extractable Fe in relation to total Fe	Fe_d_/Fe_total_
	Silt content	Silt (%)
Acidification	H^+^ concentration	10^−pH^
	Al saturation	Al_ex_/CEC_eff_
Fertility	N/P ratio	N_t_/P_total_
	Isotopic signature of total soil N	δ^15^N soil (‰ air)
	Plant available P	P_Bray_ (mg kg^−1^)
	Exchangeable base cations	Ca_ex_, K_ex_, Mg_ex_ (cmol_c_ kg^−1^)
Microorganisms	Basal respiration	(mg C kg^-1^ day^−1^)
	Metabolic quotient	qCO_2_ (µg mg^−1^C_mic_ day^-1^)
	Microbial biomass C, N, and P	C_mic_, N_mic_, P_mic_ (mg kg^−1^)
SOC	Temperature when 50% of SOC is oxidized	T50 (°C)
	Pyrophosphate extractable C	C_pyro_ (g kg^−1^)
	C in the < 63 µm fraction	C_<63 µm_ (g C kg^−1^ soil^−1^)
	C concentration within the fraction < 63 µm	C_<63 µm_ (g C kg^−1^ fraction^−1^)

Specifically, we were interested in whether weathering and acidification had an influence on fertility and/or stabilized soil organic content (SOC). This impact could be either in a direct way or indirectly by influencing microorganism. Based on this conceptual model we could formulate structural equations for fertility: ${\eta }_{1}= {\gamma }_{1}{\xi }_{1}+ {\gamma }_{2}{\xi }_{2}+ {\gamma }_{3}{\eta }_{2}+ {\varsigma }_{1}$ microorganisms: ${\eta }_{2}= {\gamma }_{3}{\xi }_{2}+ {\varsigma }_{2}$, and for stabilized SOC ${\eta }_{3}= {\gamma }_{4}{\xi }_{1}+ {\gamma }_{5}{\eta }_{2}+ {\varsigma }_{3}$, where η denotes endogeneous (dependent) latent variables, γ is the effect size, ξ exogeneous (independent) latent variables, and ς the random error term. The two exogeneous latents weathering and acidification, which are not predicted by other latents, were assumed to covary, for that we estimated a correlation matrix ϕ. In a second step, a measurement model related observed measures to the latent structure. It is given by ${y}_{i}= {\alpha }_{j}+\Lambda {\omega }_{i}+ {\epsilon }_{i}$, where ${y}_{i}$ is a vector of a measured variable, *i* is a sample index ranging from 1 to total sample size n = 39, ${\alpha }_{j}$ is a soil-pit-specific intercept, $\Lambda$ is a matrix of coefficients, ${\omega }_{i}$ is a matrix of latent variables, and ${\epsilon }_{i}$ denotes a vector of measurement errors. The SEM was fitted within a hierarchical Bayesian framework. The highly flexible approach allowed us to account for dependencies among data sampled in the same soil pit. The Bayesian estimation uses Markov Chain Monte Carlo (MCMC) algorithms to obtain multiple samples of the parameter of interest which results in a probability distribution (posterior distribution) of the latter (e.g. coefficient of a causal path between two latents). This posterior distribution allows for drawing conclusions on the uncertainty of the estimate^30^. We used the median of the distribution as effect size (ES) and report the 95% credible interval for each ES.

In this study we used *jags* for MCMC simulations. It was called from *R* with help of the package *R2jags*. Model fitting was done by three MCMC chains consisting of 50,000 iterations of which the first 30,000 were discarded. We used uninformative priors derived from the normal distribution *N* (0, 100) for all path coefficients (γ, Λ) and from the gamma distribution *Gamma* (6, 10) for error terms $\varsigma$ and $\epsilon$. In case of the land-use-specific intercept α the prior was drawn from a normal distribution *N* (0, σ) where standard deviation σ was sampled from a uniform distribution *U* (0,100). We assured convergence of MCMC chains by (i) visual interpretation of chain traces, (ii) checking the potential scale reduction factor $\widehat{R}$ to be less than 1.1, and (iii) assuring that the effective number of simulations draws for each relevant estimate exceeds 500^53^. After checking for autocorrelation among consecutive iterations in each MCMC chain, we thinned the series by retaining only every 16th sample. This led to a sample size of 3 × 1250.

## Supplementary Information

Supplementary Table S1.

Supplementary Table S2.

## Data Availability

The datasets used and/or analysed during the current study is mainly available from supplement material or otherwise from the corresponding author on reasonable request.

## References

[1] Amundson R (2021). Factors of soil formation in the 21st century. Geoderma.

[2] Richter DD (2007). Humanity’s transformation of Earth’s soil: Pedology’s new frontier. Soil Sci..

[3] Zalasiewicz J, Waters CN, Williams M (2014). Human bioturbation, and the subterranean landscape of the Anthropocene. Anthropocene.

[4] Crutzen PJ (2002). Geology of mankind. Nature.

[5] Glaser B, Birk JJ (2012). State of the scientific knowledge on properties and genesis of Anthropogenic Dark Earths in Central Amazonia (Terra Preta de Índio). Geochim. Cosmochim. Acta.

[6] Wiedner K, Schneeweiß J, Dippold MA, Glaser B (2015). Anthropogenic Dark Earth in Northern Germany—The Nordic Analogue to terra preta de Índio in Amazonia. Catena.

[7] Friend AD (2014). Carbon residence time dominates uncertainty in terrestrial vegetation responses to future climate and atmospheric CO2. Proc. Natl. Acad. Sci. U. S. A..

[8] Craig ME, Mayes MA, Sulman BN, Walker AP (2021). Biological mechanisms may contribute to soil carbon saturation patterns. Glob. Change Biol..

[9] Heitkamp F, Sylvester SP, Kessler M, Sylvester MD, Jungkunst HF (2014). Inaccessible Andean sites reveal human-induced weathering in grazed soils. Progress Phys. Geogr..

[10] Sylvester SP (2017). Relict high-Andean ecosystems challenge our concepts of naturalness and human impact. Sci. Rep..

[11] Don A, Schumacher J, Freibauer A (2011). Impact of tropical land-use change on soil organic carbon stocks—A meta-analysis. Glob. Change Biol..

[12] Dong S, Dong S, Kassam K-A, Tourrand JF, Boone RB (2016). Overview: Pastoralism in the world. Building Resilience of Human-Natural Systems of Pastoralism in the Developing. World Interdisciplinary Perspectives.

[13] Jobbagy EG, Jackson RB (2000). The vertical distribution of soil organic carbon and its relation to climate and vegetation. Ecol. Appl..

[14] Lal R (2020). Soil organic matter and water retention. Agron. J..

[15] Slessarev EW, Chadwick OA, Sokol NW, Nuccio EE, Pett-Ridge J (2022). Rock weathering controls the potential for soil carbon storage at a continental scale. Biogeochemistry.

[16] Rasmussen C (2018). Beyond clay: Towards an improved set of variables for predicting soil organic matter content. Biogeochemistry.

[17] Kramer MG, Chadwick OA (2018). Climate-driven thresholds in reactive mineral retention of soil carbon at the global scale. Nat. Clim. Change.

[18] von Fromm SF (2021). Continental-scale controls on soil organic carbon across sub-Saharan Africa. Soil.

[19] Jiménez JJ, Villar L (2017). Mineral controls on soil organic C stabilization in alpine and subalpine soils in the Central Pyrenees: Insights from wet oxidation methods, mineral dissolution treatment and radiocarbon dating. Catena.

[20] Li H (2021). Simple plant and microbial exudates destabilize mineral-associated organic matter via multiple pathways. Environ. Sci. Technol..

[21] Kleber M (2015). Minera-Organic Associations: Formation, Properties, and Relevance in Soil Environments.

[22] Mayer M (2020). Tamm Review: Influence of forest management activities on soil organic carbon stocks: A knowledge synthesis. For. Ecol. Manag..

[23] Campbell JE, Fox JF, Davis CM, Rowe HD, Thompson N (2009). Carbon and nitrogen isotopic measurements from Southern Appalachian Soils: Assessing soil carbon sequestration under climate and land-use variation. J. Environ. Eng..

[24] Masiello CA, Chadwick OA, Southon J, Torn MS, Harden JW (2004). Weathering controls on mechanisms of carbon storage in grassland soils. Global Biogeochem. Cycles.

[25] Dong S, Kassam K-A, Tourrand JF, Boone RB (2016). Building Resilience of Human-Natural Systems of Pastoralism in the Developing World. Interdisciplinary Perspectives.

[26] Sanderman J, Grandy AS (2020). Ramped thermal analysis for isolating biologically meaningful soil organic matter fractions with distinct residence times. Soil.

[27] Jungkunst HF, Goepel J, Horvath T, Ott S, Brunn M (2021). New uses for old tools: Reviving Holdridge Life Zones in soil carbon persistence research. J. Pant Nutr. Soil Sci..

[28] Craine JM (2015). Ecological interpretations of nitrogen isotope ratios of terrestrial plants and soils. Plant Soil.

[29] Goll DS, Moosdorf N, Hartmann J, Brovkin V (2014). Climate-driven changes in chemical weathering and associated phosphorus release since 1850: Implications for the land carbon balance. Geophys. Res. Lett..

[30] Grace JB (2006). Structural Equation Modeling and Natural Systems.

[31] Wagai R, Kajiura M, Asano M (2020). Iron and aluminum association with microbially processed organic matter via meso-density aggregate formation across soils: Organo-metallic glue hypothesis. Soil.

[32] Possinger AR (2020). Organo-organic and organo-mineral interfaces in soil at the nanometer scale. Nat. Commun..

[33] Reichenbach M (2021). The role of geochemistry in organic carbon stabilization against microbial decomposition in tropical rainforest soils. Soil.

[34] Doetterl S (2018). Links among warming, carbon and microbial dynamics mediated by soil mineral weathering. Nat. Geosci..

[35] Körner C (2003). Atmospheric science. Slow in, rapid out–carbon flux studies and Kyoto targets. Science.

[36] Peltzer DA (2010). Understanding ecosystem retrogression. Ecol. Monogr..

[37] Troll C (1968). Geo-Ecology of the Mountainous Regions of the Tropical Americas.

[38] Toivonen JM, Kessler M, Ruokolainen K, Hertel D (2011). Accessibility predicts structural variation of Andean Polylepis forests. Biodivers. Conserv..

[39] Chepstow-Lusty AJ (2009). Putting the rise of the Inca Empire within a climatic and land management context. Clim. Past.

[40] Reeuwijk LP (2002). Procedures of Soil Analysis.

[41] Lüer B, Böhmer A (2000). Vergleich zwischen Perkolation und Extraktion mit 1M NH4Cl-Lösung zur Bestimmung der effektiven Kationenaustauschkapazität (KAKeff) von Böden. J. Pant Nutr. Soil Sci..

[42] Blume H-P, Stahr K, Leinweber P (2011). Bodenkundliches Praktikum. Eine Einführung in pedologisches Arbeiten für Ökologen, Land- und Forstwirte, Geo- und Umweltwissenschaftler.

[43] Fox PM, Nico PS, Tfaily MM, Heckman K, Davis JA (2017). Characterization of natural organic matter in low-carbon sediments: Extraction and analytical approaches. Org. Geochem..

[44] Bray RH, Kurtz LT (1945). Determination of total, organic, and available forms of phosphorus in soils. Soil Sci..

[45] Diochon A, Gillespie AW, Ellert BH, Janzen HH, Gregorich EG (2016). Recovery and dynamics of decomposing plant residue in soil: An evaluation of three fractionation methods. Eur. J. Soil Sci..

[46] Minasny B, McBratney AB, Wadoux AM-C, Akoeb EN, Sabrina T (2020). Precocious 19th century soil carbon science. Geoderma Reg..

[47] Marin-Spiotta E (2014). Long-term stabilization of deep soil carbon by fire and burial during early Holocene climate change. Nat. Geosci..

[48] Fernández JM, Peltre C, Craine JM, Plante AF (2012). Improved characterization of soil organic matter by thermal analysis using CO_2_/H_2_O evolved gas analysis. Environ. Sci. Technol..

[49] Vuong TX, Heitkamp F, Jungkunst HF, Reimer A, Gerold G (2013). Simultaneous measurement of soil organic and inorganic carbon: Evaluation of a thermal gradient analysis. J Soils Sediments.

[50] Anderson T, Domsch K (1993). The metabolic quotient for CO_2_ (qCO_2_) as a specific activity parameter to assess the effects of environmental conditions, such as ph, on the microbial biomass of forest soils. Soil Biol. Biochem..

[51] Vance ED, Brookes PC, Jenkinson DS (1987). An extraction method for measuring soil microbial biomass C. Soil Biol. Biochem..

[52] Brookes PC, Landman A, Pruden G, Jenkinson DS (1985). Chloroform fumigation and the release of soil nitrogen: A rapid direct extraction method to measure microbial biomass nitrogen in soil. Soil Biol. Biochem..

[53] Gelman A (2014). Bayesian Data Analysis.

