# Supplementary material for: Land-use induced soil carbon stabilization at the expense of rock derived nutrients: insights from pristine Andean soils
Source: Sci Rep. 2023 Mar 20;13:4584. doi: 10.1038/s41598-023-30801-x (PMC10027661; doi:10.1038/s41598-023-30801-x)
Supplement: Supplementary file 1 — Supplementary Table S1. [file 41598_2023_30801_MOESM1_ESM.docx]

**Table-S-1:** Mean values of soil parameters under investigation. PF, pristine forests; PG, pristine grasslands; MP, managed pastures

|  | Profile | Depth | Soil mass  (≤ 2mm) | Coarse fragments | Sand | Silt | Clay | Bulk density | Fine roots | SOC | N_t_ |  |
| --- | --- | --- | --- | --- | --- | --- | --- | --- | --- | --- | --- | --- |
|  |  | (cm) | (kg m^-2^) | (wgt-%) | (%) | (%) | (%) | (g cm^-3^) | (g kg^-1^) | (g kg^-1^) | (g kg^-1^) | |
|  |  |  |  |  |  |  |  |  |  |  |  | |
| PF | 1 | 5 | 5.38 | 39.03 |  |  |  | 0.07 | 556.71 | 295.7 | 16.18 | |
| PF | 1 | 10 | 12.05 | 0 | 69.02 | 28.67 | 2.31 | 0.24 | 50.23 | 224.78 | 13.4 |  |
| PF | 1 | 20 | 37.84 | 22.86 | 72.91 | 26.48 | 0.61 | 0.38 | 41.61 | 145.07 | 8.93 |  |
| PF | 2 | 5 | 18.22 | 0 | 72.79 | 25.87 | 1.34 | 0.36 | 30.79 | 162.7 | 10.17 |  |
| PF | 2 | 10 | 25.47 | 43.91 | 85.20 | 13.50 | 1.30 | 0.41 | 35.31 | 103.15 | 7.23 |  |
| PF | 2 | 20 | 62.05 | 71.55 | 88.11 | 11.09 | 0.81 | 0.43 | 22.41 | 123.79 | 7.21 |  |
| PF | 2 | 30 | 73.95 | 68.92 | 78.70 | 19.91 | 1.39 | 0.43 | 34.11 | 68.97 | 4.58 |  |
| PF | 3 | 5 | 18.81 | 2.79 | 84.33 | 14.80 | 0.87 | 0.37 | 37.35 | 180.01 | 10.38 |  |
| PF | 3 | 10 | 22.08 | 12.02 | 82.36 | 16.33 | 1.32 | 0.42 | 25.37 | 170.46 | 9.79 |  |
| PF | 3 | 20 | 49.25 | 21.18 | 76.43 | 21.52 | 2.05 | 0.46 | 22.29 | 95.8 | 5.86 |  |
| PF | 3 | 30 | 85.28 | 65.5 | 73.35 | 24.82 | 1.83 | 0.74 | 16.26 | 65.21 | 4.34 |  |
| PF | 3 | 40 | 47.62 | 64.87 | 50.86 | 45.14 | 4.00 | 0.33 | 2.58 | 55.45 | 3.77 |  |
| PG | 1 | 5 | 20.36 | 6.26 | 76.79 | 21.89 | 1.32 | 0.4 | 32.64 | 160.09 | 11.35 |  |
| PG | 1 | 10 | 18.6 | 6.55 | 69.56 | 23.54 | 6.90 | 0.36 | 21.62 | 139.2 | 10.83 |  |
| PG | 1 | 20 | 30.19 | 11.05 | 55.70 | 41.26 | 3.04 | 0.58 | 15.21 | 111.29 | 7.62 |  |
| PG | 2 | 5 | 20.52 | 3.32 | 73.00 | 25.42 | 1.58 | 0.4 | 21.17 | 125.33 | 9.56 |  |
| PG | 2 | 10 | 25.32 | 31.75 | 71.66 | 27.27 | 1.07 | 0.45 | 26.96 | 97.86 | 7.77 |  |
| PG | 2 | 20 | 41.44 | 84.77 | 66.79 | 30.99 | 2.23 | 0.3 | 19.72 | 74.82 | 5.32 |  |
| PG | 2 | 30 | 52.25 | 84.23 | 75.43 | 24.06 | 0.51 | 0.32 | 6.39 | 58.45 | 4.23 |  |
| PG | 2 | 40 | 69.9 | 76.41 | 69.48 | 29.41 | 1.11 | 0.32 | 16.99 | 58.27 | 4.16 |  |
| PG | 3 | 5 | 26.7 | 9.99 | 79.20 | 19.82 | 0.98 | 0.52 | 15.42 | 114.7 | 8.45 |  |
| PG | 3 | 10 | 23.82 | 10.85 | 69.51 | 28.76 | 1.72 | 0.46 | 14.69 | 98.81 | 7.65 |  |
| PG | 3 | 20 | 57.18 | 43.37 | 59.22 | 38.79 | 1.99 | 0.47 | 22.7 | 99.71 | 6.92 |  |
| PG | 3 | 30 | 34.68 | 67.92 | 70.65 | 28.02 | 1.33 | 0.21 | 6.03 | 100.1 | 6.43 |  |
| PG | 3 | 40 | 71.54 | 79.85 | 71.79 | 27.10 | 1.11 | 0.58 | 15.51 | 95.03 | 6.57 |  |
| MP | 1 | 5 | 36.41 | 18.87 | 36.16 | 62.11 | 1.73 | 0.7 | 10.16 | 115.57 | 7.98 |  |
| MP | 1 | 10 | 35.78 | 38.22 | 52.39 | 46.55 | 1.06 | 0.63 | 12.45 | 104.54 | 7.46 |  |
| MP | 1 | 20 | 71.43 | 0 | 53.92 | 45.07 | 1.01 | 0.71 | 1.5 | 99.67 | 6.48 |  |
| MP | 1 | 30 | 44.79 | 75.64 | 67.39 | 31.88 | 0.73 | 0.28 | 3.93 | 72.86 | 4.86 |  |
| MP | 1 | 40 | 52.47 | 78.73 | 72.25 | 27.01 | 0.75 | 0.36 | 0.59 | 55.41 | 3.82 |  |
| MP | 2 | 5 | 31.12 | 8.46 | 51.49 | 47.60 | 0.90 | 0.61 | 16.02 | 139.35 | 9.02 |  |
| MP | 2 | 10 | 31.06 | 12.9 | 59.35 | 40.07 | 0.58 | 0.6 | 15.34 | 129.97 | 8.23 |  |
| MP | 2 | 20 | 61.29 | 27.5 | 49.55 | 49.17 | 1.27 | 0.57 | 3.33 | 112.09 | 6.16 |  |
| MP | 2 | 30 | 49.52 | 46.22 | 63.21 | 35.54 | 1.25 | 0.41 | 1.9 | 94.37 | 5.39 |  |
| MP | 2 | 40 | 40.68 | 0 | 64.58 | 34.37 | 1.04 | 0.41 | 0 | 68.95 | 3.99 |  |
| MP | 3 | 5 | 32.17 | 16.87 | 60.86 | 36.15 | 2.99 | 0.61 | 44.33 | 150.91 | 13.86 |  |
| MP | 3 | 10 | 35.45 | 21.95 | 61.56 | 37.51 | 0.93 | 0.67 | 12.11 | 120.16 | 11.74 |  |
| MP | 3 | 20 | 68.6 | 37.12 | 64.40 | 34.30 | 1.30 | 0.6 | 1.5 | 102.1 | 6.28 |  |
| MP | 3 | 30 | 59.95 | 48.07 | 69.78 | 29.33 | 0.89 | 0.6 | 0.69 | 76.72 | 4.5 |  |

SOC, soil organic carbon; N_t_, total nitrogen, blank cells: not enough material for analysis

**Table-S-1**: continuation

|  | Profile | Depth | C_mic_ | N_mic_ | qCO_2_ | C_<63µm_ | C_<63µm_ | δ^13^C soil | δ^13^C roots* | C_pyro_ | | T50 |  |  |
| --- | --- | --- | --- | --- | --- | --- | --- | --- | --- | --- | --- | --- | --- | --- |
|  |  | (cm) | (mg kg^-1^) | (mg kg^-1^) | (µg mg^-1^C_mic_ day^-1^) | g C kg^-1^ fraction^-1^ | g C kg^-1^ soil^-1^ | (‰ V-PDB) | (‰ air) | (g kg^-1^) | | (°C) |  |  |
|  |  |  |  |  |  |  | | |  | |  | | |  |
| PF | 1 | 5 | 3398 | 396 |  |  |  |  |  | 91.96 | | 299 |  |  |
| PF | 1 | 10 | 3343 | 462 | 17.99 | 99.38 | 23.74 | -25.915 |  | 69.91 | | 306 |  |  |
| PF | 1 | 20 | 2861 | 412 | 25.46 | 77.44 | 18.36 | -25.478 | -27.644 | 46.19 | | 297 |  |  |
| PF | 2 | 5 | 3558 | 487 | 16.9 | 159.44 | 47.87 | -23.626 |  | 45.30 | | 314 |  |  |
| PF | 2 | 10 | 2450 | 366 | 29.77 | 76.78 | 8.64 | -25.228 |  | 24.15 | | 297 |  |  |
| PF | 2 | 20 | 2447 | 379 | 28.66 | 76.58 | 7.59 | -25.635 |  | 35.24 | | 295 |  |  |
| PF | 2 | 30 | 1460 | 231 | 30.38 | 59.18 | 12.33 | -25.025 | -27.424 | 16.09 | | 296 |  |  |
| PF | 3 | 5 | 1998 | 315 | 47.72 | 106.91 | 13.17 | -25.978 |  | 47.90 | | 311 |  |  |
| PF | 3 | 10 | 1651 | 286 | 78.9 | 110.19 | 15.85 | -26.261 |  | 47.39 | | 298 |  |  |
| PF | 3 | 20 | 1555 | 243 | 22.2 | 75.78 | 16.99 | -25.486 |  | 22.60 | | 294 |  |  |
| PF | 3 | 30 | 1204 | 198 | 18.79 | 65.48 | 17.40 | -25.117 |  | 13.82 | | 293 |  |  |
| PF | 3 | 40 | 798.55 | 132 | 37.78 | 64.18 | 31.67 | -24.845 | -27.575 | 11.09 | | 287 |  |  |
| PG | 1 | 5 | 3752 | 530 | 33 | 110.78 | 23.45 | -24.987 |  | 61.17 | | 292 |  |  |
| PG | 1 | 10 | 4239 | 578 | 25.65 | 88.70 | 26.01 | -24.662 |  | 50.18 | | 294 |  |  |
| PG | 1 | 20 | 2059 | 352 | 37.63 | 91.90 | 39.61 | -24.861 | -26.359 | 32.79 | | 297 |  |  |
| PG | 2 | 5 | 3791 | 620 | 15.52 | 74.06 | 17.97 | -25.017 |  | 32.26 | | 289 |  |  |
| PG | 2 | 10 | 2608 | 396 | 35.26 | 67.73 | 17.63 | -24.766 |  | 23.37 | | 303 |  |  |
| PG | 2 | 20 | 1580 | 281 | 8.22 | 69.20 | 23.22 | -24.574 |  | 16.48 | | 327 |  |  |
| PG | 2 | 30 | 1290 | 215 |  | 68.83 | 17.29 | -24.478 |  | 12.87 | | 304 |  |  |
| PG | 2 | 40 | 594 | 99 |  | 72.91 | 22.48 | -24.295 | -26.825 | 17.15 | | 298 |  |  |
| PG | 3 | 5 | 2045 | 256 | 31.95 | 90.98 | 20.11 | -24.897 |  | 43.00 | | 287 |  |  |
| PG | 3 | 10 | 1725 | 219 |  | 95.33 | 27.99 | -24.772 |  | 34.91 | |  |  |  |
| PG | 3 | 20 | 1785 | 235 | 9.47 | 91.06 | 36.15 | -24.693 |  | 34.66 | | 298 |  |  |
| PG | 3 | 30 | 1175 | 154 | 21.69 | 98.59 | 29.02 | -24.411 |  | 50.80 | | 298 |  |  |
| PG | 3 | 40 | 1047 | 117 | 39.66 | 103.80 | 28.56 | -24.026 | -26.355 | 59.02 | | 296 |  |  |
| MP | 1 | 5 | 897 | 90 |  | 68.12 | 43.19 | -24.783 |  | 61.61 | | 329 |  |  |
| MP | 1 | 10 | 796 | 78 | 18.82 | 131.11 | 66.97 | -24.549 |  | 54.02 | | 348 |  |  |
| MP | 1 | 20 | 489 | 43 | 27.02 | 133.75 | 67.35 | -24.500 |  | 51.85 | | 344 |  |  |
| MP | 1 | 30 | 220 | 19 | 373.96 | 131.42 | 48.16 | -24.343 |  | 37.90 | | 354 |  |  |
| MP | 1 | 40 | 92 | 6 | 107.99 | 105.59 | 32.35 | -24.032 | -25.947 | 28.82 | | 333 |  |  |
| MP | 2 | 5 | 1780 | 206 | 46.45 | 140.22 | 68.29 | -24.831 |  | 85.05 | | 338 |  |  |
| MP | 2 | 10 | 866 | 110 | 51.3 | 143.76 | 59.81 | -24.719 |  | 79.32 | | 343 |  |  |
| MP | 2 | 20 | 458 | 52 | 278 | 136.02 | 72.24 | -24.615 |  | 65.94 | | 352 |  |  |
| MP | 2 | 30 | 204 | 26 | 112.6 | 131.55 | 53.02 | -24.605 |  | 53.82 | | 308 |  |  |
| MP | 2 | 40 | 22 | 6 |  | 116.24 | 44.82 | -24.538 | -25.609 | 33.40 | | 403 |  |  |
| MP | 3 | 5 | 2483 | 284 | 14.53 | 136.64 | 51.33 | -24.895 |  | 76.95 | | 347 |  |  |
| MP | 3 | 10 | 1211 | 132 | 60.37 | 151.81 | 62.84 | -24.512 |  | 61.69 | | 333 |  |  |
| MP | 3 | 20 | 488 | 62 | 136.65 | 145.13 | 57.48 | -24.537 |  | 51.93 | | 360 |  |  |
| MP | 3 | 30 | 192 | 25 | 201.17 | 132.21 | 46.09 | -24.517 | -25.972 | 33.22 | | 322 |  |  |

|  |  |  |  |  |  |  |  |  |  |  |  |
| --- | --- | --- | --- | --- | --- | --- | --- | --- | --- | --- | --- |

C_mic_, microbial biomass carbon; N_mic_, microbial biomass nitrogen; qCO_2_, metabolic quotient; C_<63µm_, C concentration within the texture fraction <63 µm; MAOM_soil_, C concentration of the texture fraction <63µm related to bulk soil; C_pyro_, pyrophosphate extractable carbon; δ^13^C roots*, the roots of all depths were combined for the delta δ^13^C determination

**Table-S-1**: continuation

|  | Profile | Depths | δ^15^N soil | Al_total_ | Al_o_ | Fe_total_ | Fe_o_ | Fe_d_ | pH | P_bray_ |
| --- | --- | --- | --- | --- | --- | --- | --- | --- | --- | --- |
|  |  | (cm) | (‰ air) | (g kg^-1^) | (g kg^-1^) | (g kg^-1^) | (g kg^-1^) | (g kg^-1^) | CaCl_2_ | (mg kg^-1^) |
|  |  |  |  |  |  |  |  |  |  |  |
| PF | 1 | 5 |  | 28.82 | 0.73 | 19.17 | 1.77 | 5.5 | 5.1 | 55.92 |
| PF | 1 | 10 | 1.147 | 40.04 | 0.87 | 24.81 | 2.11 | 5.5 | 5.1 | 33.21 |
| PF | 1 | 20 | 2.401 | 52.41 | 1.37 | 32.55 | 3.58 | 9.08 | 4.7 | 25.88 |
| PF | 2 | 5 | 1.627 | 51.37 | 1.01 | 27.57 | 2 | 9.13 | 4.4 | 3.36 |
| PF | 2 | 10 | 3.139 | 60.93 | 0.96 | 32.04 | 2.06 | 6.21 | 4.5 | 5.75 |
| PF | 2 | 20 | 2.270 | 57.82 | 0.87 | 30.23 | 1.62 | 7.01 | 4.4 | 0.74 |
| PF | 2 | 30 | 3.705 | 67.64 | 1.3 | 34.21 | 2.65 | 6.56 | 4.4 | 1.71 |
| PF | 3 | 5 | 0.772 | 52.01 | 1.01 | 26.47 | 2.06 | 5.34 | 4.8 | 43.14 |
| PF | 3 | 10 | 0.548 | 52.00 | 0.92 | 25.67 | 1.84 | 6.32 | 4.9 | 19.88 |
| PF | 3 | 20 | 2.778 | 62.52 | 1.32 | 33.27 | 2.66 | 6.52 | 4.6 | 1.02 |
| PF | 3 | 30 | 4.074 | 67.76 | 1.37 | 36.03 | 2.82 | 7.99 | 4.5 | 1.04 |
| PF | 3 | 40 | 4.761 | 69.72 | 1.65 | 36.13 | 3.17 | 8.31 | 4.4 | 1.63 |
| PG | 1 | 5 | 1.288 | 46.79 | 1.38 | 22.38 | 2.28 | 5.42 | 4.6 | 27.64 |
| PG | 1 | 10 | 1.871 | 50.63 | 1.62 | 21.48 | 2.56 | 6 | 4.5 | 22.93 |
| PG | 1 | 20 | 2.554 | 56.29 | 1.7 | 25.78 | 2.82 | 6.84 | 4.6 | 1.75 |
| PG | 2 | 5 | 3.406 | 54.31 | 0.61 | 32.38 | 1.18 | 6.84 | 4.4 | 29.76 |
| PG | 2 | 10 | 4.317 | 58.29 | 1.25 | 36 | 2.37 | 9.96 | 4.2 | 23.19 |
| PG | 2 | 20 | 5.280 | 63.54 | 2.08 | 40.56 | 3.48 | 7.97 | 4.2 | 4.46 |
| PG | 2 | 30 | 5.685 | 68.32 | 2.16 | 42.72 | 4.19 | 11.89 | 4.1 | 5.98 |
| PG | 2 | 40 | 5.935 | 69.70 | 3.41 | 45.22 | 6.41 | 12.08 | 4.2 | 9.48 |
| PG | 3 | 5 | 3.166 | 56.81 | 1.75 | 36.16 | 4.3 | 9.27 | 4.1 | 22.51 |
| PG | 3 | 10 | 3.723 | 60.52 | 1.93 | 36.26 | 4.49 | 11.54 | 4.0 | 26.06 |
| PG | 3 | 20 | 3.733 | 60.18 | 2.38 | 37.67 | 5.15 | 9.72 | 3.9 | 5.32 |
| PG | 3 | 30 | 4.180 | 64.26 | 3.99 | 40.73 | 6.35 | 11.1 | 4.0 | 11.49 |
| PG | 3 | 40 | 4.668 | 69.23 | 6.85 | 43.16 | 9.7 | 12.57 | 4.0 | 11.49 |
| MP | 1 | 5 | 4.771 | 65.72 | 8.7 | 32.62 | 7.5 | 12.91 | 4.1 | 6.64 |
| MP | 1 | 10 | 5.640 | 70.80 | 10.15 | 32.88 | 8.15 | 13.76 | 4.1 | 4 |
| MP | 1 | 20 | 6.169 | 73.25 | 8.42 | 33.73 | 5.54 | 10.38 | 4.2 | 7.12 |
| MP | 1 | 30 | 5.328 | 82.79 | 8.15 | 32.21 | 3.57 | 10.94 | 4.5 | 10.82 |
| MP | 1 | 40 | 4.945 | 85.37 | 7.28 | 36.42 | 2.62 | 6.65 | 4.6 | 24.74 |
| MP | 2 | 5 | 4.634 | 57.06 | 4.25 | 29.07 | 3.74 | 9.3 | 4.1 | 9.03 |
| MP | 2 | 10 | 5.978 | 63.65 | 10.55 | 31.37 | 7.15 | 12.95 | 4.1 | 5.61 |
| MP | 2 | 20 | 6.966 | 68.84 | 10.95 | 31.89 | 6.25 | 10.67 | 4.1 | 1.98 |
| MP | 2 | 30 | 7.186 | 76.15 | 12.09 | 31.86 | 5.09 | 9.7 | 4.2 | 3.9 |
| MP | 2 | 40 | 6.909 | 82.20 | 11.6 | 32.15 | 3.02 | 6.55 | 4.4 | 4.97 |
| MP | 3 | 5 | 4.834 | 56.85 | 5.4 | 55.76 | 8.9 | 20.54 | 4.1 | 11.21 |
| MP | 3 | 10 | 6.230 | 66.39 | 7.7 | 63.47 | 8.2 | 23.34 | 4.0 | 6.38 |
| MP | 3 | 20 | 6.731 | 68.81 | 8.15 | 63.11 | 5.85 | 21.38 | 4.0 | 2.58 |
| MP | 3 | 30 | 6.990 | 76.55 | 9.37 | 66.16 | 4.73 | 18.93 | 4.2 | 4.98 |

A_total_, total aluminium; Al_o_, oxalate-extractable aluminium; Fe_total_, total iron; Fe_o_, oxalate-extractable iron;
Fe_d_, dithionite-extractable iron; P_Bray_, plant available phosphorus

**Table-S-1**: continuation

|  | Profile | Depth | Al_ex_ | Ca_ex_ | Fe_ex_ | K_ex_ | Mg_ex_ | Mn_ex_ | Na_ex_ | H_ex_ | CEC_eff_ |
| --- | --- | --- | --- | --- | --- | --- | --- | --- | --- | --- | --- |
|  |  | cm | ---------------------------------------------- (cmol_c_ kg^-1^) ------------------------------------------------------------------ | | | | | | | | |
|  |  |  |  |  |  |  |  |  |  |  |  |
| PF | 1 | 5 | 0.06 | 35.29 | 0 | 0.91 | 18.35 | 1.38 | 0.42 | 0 | 56.42 |
| PF | 1 | 10 | 0 | 2.96 | 0 | 0.09 | 1.59 | 0.16 | 0.02 | 0 | 4.82 |
| PF | 1 | 20 | 0.04 | 22.78 | 0 | 0.64 | 9.32 | 1.1 | 0 | 0 | 33.87 |
| PF | 2 | 5 | 0.12 | 20.23 | 0 | 0.78 | 6.87 | 2.08 | 0.17 | 0.03 | 30.27 |
| PF | 2 | 10 | 0.18 | 11.74 | 0 | 0.5 | 5.06 | 1.6 | 0.07 | 0.03 | 19.18 |
| PF | 2 | 20 | 0.15 | 14.57 | 0 | 0.44 | 5.29 | 0.98 | 0.1 | 0.07 | 21.6 |
| PF | 2 | 30 | 0.39 | 7.45 | 0 | 0.31 | 3.41 | 0.82 | 0.08 | 0.04 | 12.5 |
| PF | 3 | 5 | 0.05 | 26.63 | 0 | 0.66 | 9.61 | 1.45 | 0.22 | 0 | 38.63 |
| PF | 3 | 10 | 0.04 | 29.2 | 0 | 0.6 | 10.5 | 1.34 | 0.1 | 0 | 41.77 |
| PF | 3 | 20 | 0.09 | 12.99 | 0 | 0.42 | 5.07 | 0.7 | 0.06 | 0 | 19.33 |
| PF | 3 | 30 | 0.3 | 6.79 | 0 | 0.19 | 3.28 | 0.53 | 0 | 0.02 | 11.11 |
| PF | 3 | 40 | 0.66 | 4.62 | 0 | 0.14 | 2.48 | 0.38 | 0.01 | 0.04 | 8.33 |
| PG | 1 | 5 | 0.16 | 11.71 | 0 | 1.04 | 6.8 | 5.36 | 0.2 | 0 | 25.27 |
| PG | 1 | 10 | 0.19 | 8.46 | 0 | 0.88 | 5.11 | 4.16 | 0.11 | 0 | 18.9 |
| PG | 1 | 20 | 0.2 | 13.6 | 0 | 0.54 | 3.79 | 1.6 | 0.06 | 0.01 | 19.81 |
| PG | 2 | 5 | 0.12 | 8.48 | 0 | 0.74 | 4.02 | 1.16 | 0.03 | 0.04 | 14.58 |
| PG | 2 | 10 | 0.48 | 5.17 | 0 | 0.47 | 2.6 | 0.88 | 0.02 | 0.1 | 9.72 |
| PG | 2 | 20 | 1.43 | 3.44 | 0 | 0.25 | 1.61 | 0.3 | 0.04 | 0.11 | 7.18 |
| PG | 2 | 30 | 2.07 | 2.5 | 0 | 0.22 | 1.36 | 0.19 | 0.02 | 0.12 | 6.48 |
| PG | 2 | 40 | 2.69 | 2.85 | 0 | 0.2 | 1.37 | 0.2 | 0.02 | 0.1 | 7.43 |
| PG | 3 | 5 | 1.6 | 6.68 | 0.09 | 0.4 | 2.23 | 0.76 | 0.01 | 0.26 | 12.02 |
| PG | 3 | 10 | 1.96 | 5.18 | 0.11 | 0.34 | 1.78 | 0.5 | 0.03 | 0.22 | 10.11 |
| PG | 3 | 20 | 2.69 | 4.61 | 0.01 | 0.32 | 1.83 | 0.34 | 0.01 | 0.24 | 10.05 |
| PG | 3 | 30 | 3.86 | 4.65 | 0.24 | 0.32 | 1.41 | 0 | 0.03 | 0.15 | 10.65 |
| PG | 3 | 40 | 5.57 | 2.88 | 0.3 | 0.17 | 0.99 | 0 | 0 | 0.13 | 10.04 |
| MP | 1 | 5 | 5.44 | 1.64 | 0 | 0.14 | 0.48 | 0.16 | 0 | 0.11 | 7.97 |
| MP | 1 | 10 | 5.4 | 1.11 | 0 | 0.16 | 0.38 | 0.1 | 0.03 | 0.09 | 7.26 |
| MP | 1 | 20 | 4.33 | 1.73 | 0 | 0.08 | 0.4 | 0 | 0 | 0.05 | 6.6 |
| MP | 1 | 30 | 2.33 | 0.8 | 0 | 0.03 | 0.13 | 0 | 0 | 0.01 | 3.31 |
| MP | 1 | 40 | 1.23 | 0.73 | 0 | 0.39 | 0.13 | 0 | 0 | 0 | 2.49 |
| MP | 2 | 5 | 6.17 | 1.6 | 0 | 0.3 | 0.63 | 0.34 | 0.14 | 0.11 | 9.29 |
| MP | 2 | 10 | 6.97 | 1.3 | 0 | 0.24 | 0.56 | 0.07 | 0.1 | 0.1 | 9.34 |
| MP | 2 | 20 | 5.99 | 0.71 | 0 | 0.09 | 0.2 | 0 | 0.04 | 0.06 | 7.09 |
| MP | 2 | 30 | 4.73 | 0.53 | 0 | 0.03 | 0.1 | 0 | 0.03 | 0.03 | 5.46 |
| MP | 2 | 40 | 3.21 | 0.44 | 0 | 0.02 | 0.04 | 0 | 0 | 0.01 | 3.72 |
| MP | 3 | 5 | 4.92 | 2.09 | 0 | 0.38 | 0.61 | 1.1 | 0.06 | 0.15 | 9.32 |
| MP | 3 | 10 | 6.07 | 1.94 | 0 | 0.22 | 0.3 | 0.17 | 0.07 | 0.11 | 8.88 |
| MP | 3 | 20 | 6.22 | 0.51 | 0 | 0.1 | 0.01 | 0 | 0 | 0.12 | 6.97 |
| MP | 3 | 30 | 5.38 | 0.39 | 0 | 0.05 | 0.06 | 0 | 0 | 0.05 | 5.93 |
