# Supplementary material for: Land-use induced soil carbon stabilization at the expense of rock derived nutrients: insights from pristine Andean soils
Source: Sci Rep. 2023 Mar 20;13:4584. doi: 10.1038/s41598-023-30801-x (PMC10027661; doi:10.1038/s41598-023-30801-x)
Supplement: Supplementary file 2 — Supplementary Table S2. [file 41598_2023_30801_MOESM2_ESM.docx]

**Table S-2:** Mean value or stocks (SD) of soil parameters under investigation. Lower case letters indicate significant differences between mean values or stocks (p < 0.05).

| Parameter | unit | PF | PG | MP | F-value | p-value |
| --- | --- | --- | --- | --- | --- | --- |
| Soil mass | (kg m^-2^) | 153 (50) | 164 (48) | 217 (13) | 0.71 | 0.529 |
| SOC | (kg m^-2^) | 16.1 (3.2) | 15.3 (3.5) | 21.6 (0.6) | 1.531 | 0.290 |
| Root biomass | (kg m^-2^) | 4.8 (0.5)^a^ | 2.8 (0.6)^ab^ | 1.5 (0.3)^b^ | 12.24 | 0.008** |
| C associated with the fine mineral fraction (<63 µm) | (kg m^-2^) | 36.8 (14.8) | 50.3 (13.4) | 87.5 (9.9) | 4.16 | 0.074^+^ |
| Δδ^13^C (difference between plant root material and C_<63 µm_) | (‰ V-PDB) | -2.10 (0.12)^a^ | -1.92 (0.23)^ab^ | -1.29 (0.17)^b^ | 5.68 | 0.041* |
| C_<63 µm_ (C concentrations in the soil fraction <63 µm sometimes referred to as MAOM) | (kg m^-2^) | 2.6 (1.0)^a^ | 4.2 (1.2)^a^ | 12.0 (0.7)^b^ | 25.53 | 0.001** |
| C_<63 µm_ | (% of SOC) | 15.2 (3.6)^a^ | 26.9 (1.8)^a^ | 55.7 (2.5)^b^ | 58.82 | < 0.001*** |
| C-concentration in <63 µm | (g kg^-1^ fraction^-1^) | 76.0 (1.0)^a^ | 88.1 (8.7)^a^ | 130.3 (7.1)^b^ | 19.15 | 0.003** |
| T50 | (°C) | 297 (2)^a^ | 288 (13)^a^ | 344 (2)^b^ | 16.12 | 0.004** |
| C_Pyro_ (Pyrophosphate extractable C) | (kg m^-2^) | 4.1 (0.5)^a^ | 5.7 (2.2)^a^ | 11.4 (0.9)^b^ | 7.579 | 0.023* |
| C_Pyro_ | (% of SOC) | 26.6 (2.7)^a^ | 35.3 (6.3)^ab^ | 52.6 (2.4)^b^ | 9.916 | 0.013* |
| Fe_o_ (Oxalate extractable Fe) | (kg m^-2^) | 0.39 (0.13) | 0.83 (0.36) | 1.21 (0.06) | 3.372 | 0.104 |
| Al_o_ (Oxalate extractable Al) | (kg m^-2^) | 0.19 (0.07)^a^ | 0.48 (0.22)^a^ | 1.93 (0.19)^b^ | 30.16 | < 0.001*** |
| pH (CaCl_2_) |  | 4.6 (0.1) | 4.2 (0.2) | 4.2 (0.1) | 2.91 | 0.131 |
| Al saturation | (% of CEC) | 1.0 (0.5)^a^ | 19.2 (9.9)^a^ | 74.6 (3.9)^b^ | 38.84 | < 0.001*** |
| Extractable Al | (mol_c_ m^-2^) | 3.7 (1.8)^a^ | 38.6 (22.0)^a^ | 104.4 (8.8)^b^ | 13.89 | 0.006** |
| Base saturation | (% of CEC) | 94.5 (1.3)^a^ | 71.6 (6.9)^b^ | 22.8 (4.3)^c^ | 59.86 | < 0.001*** |
| Extractable bases | (mol_c_ m^-2^) | 279 (63)^a^ | 126 (3.3)^a^ | 31 (5)^b^ | 11.71 | 0.009** |
| CEC_eff_ | (mol_c_ m^-2^) | 296 (69) | 180 (23) | 140 (5) | 3.813 | 0.085^+^ |
| C_mic_ | (g m^-2^) | 281 (64) | 283 (33) | 133 (18) | 4.077 | 0.076^+^ |
| C_mic_ | (% of SOC) | 1.75 (0.19)^a^ | 1.97 (0.26)^a^ | 0.62 (0.10)^b^ | 13.7 | 0.006** |
| qCO_2_ | (mg CO_2_-C kg^-1^ C_mic_^-1^ day^-1^) | 27.2 (3.1)^a^ | 21.3 (7.5)^a^ | 115.5 (5.6)^b^ | 83.68 | < 0.001*** |
| N_mic_ | (mg kg^-1^) | 321 (61) | 300 (85) | 70 (17) | 5.192 | 0.049* |
| N_mic_ | (g m^-2^) | 43.2 (10.5) | 41.3 (6.0) | 14.8 (2.7) | 4.943 | 0.054^+^ |
| N_mic_ | (% of N_total_) | 4.3 (0.4) | 4.1 (0.7) | 1.0 (0.2) | 14.15 | 0.005** |
|  |  |  |  |  |  |  |
| N_total_ | (kg m^-2^) | 1.00 (0.21) | 1.10 (0.24) | 1.45 (0.06) | 1.602 | 0.277 |
| δ^15^N | (‰ air) | 2.71 (0.42)^a^ | 3.78 (0.96)^ab^ | 6.13 (0.34)^b^ | 7.544 | 0.023* |
| P_bray_ | (g m^-2^) | 1.2 (0.4) | 2.1 (0.5) | 1.6 (0.6) | 0.777 | 0.501 |
